# Protective effects of ascorbic acid and calcitriol combination on airway remodelling in ovalbumin-induced chronic asthma

**DOI:** 10.1080/13880209.2019.1710218

**Published:** 2020-01-16

**Authors:** Farzaneh Kianian, Seyed Morteza Karimian, Mehri Kadkhodaee, Nasrin Takzaree, Behjat Seifi, Hamid Reza Sadeghipour

**Affiliations:** a Department of Physiology, School of Medicine, Tehran University of Medical Sciences, Tehran, Iran; b Department of Anatomy and Histology, School of Medicine, Tehran University of Medical Sciences, Tehran, Iran

**Keywords:** Oxidative stress, inflammation, interleukin 13, immunoglobulin E, goblet hyperplasia, subepithelial fibrosis

## Abstract

**Context:**

Airway remodelling is one of the most refractory problems in asthma. According to the critical roles of oxidative stress and inflammation in airway remodelling, it is supposed that ascorbic acid and calcitriol have beneficial effects. However, a combination of antioxidants may be more effective for asthma therapy.

**Objective:**

This study investigated the protective effects of ascorbic acid in combination with calcitriol on airway remodelling in ovalbumin (OVA)-induced chronic asthma.

**Materials and methods:**

BALB/c mice were assigned into seven groups: (1) Control; (2) Asthma; (3) Ineffective C (orally 39 mg/kg ascorbic acid); (4) Ineffective D (intraperitoneally 1.5 μg/kg calcitriol); (5) Effective C (orally 130 mg/kg ascorbic acid); (6) Effective D (intraperitoneally 5 μg/kg calcitriol); (7) Combination (orally 39 mg/kg ascorbic acid + intraperitoneally 1.5 μg/kg calcitriol). All animals were sensitized and challenged with OVA except in the control group (normal saline). In all treatment groups, mice were administrated vitamins 30 min before each challenge (three times per week for 8 consecutive weeks).

**Results:**

In comparison with the asthma group, co-administration of ineffective doses of ascorbic acid and calcitriol led to the decreased levels of IL-13 (50.5 ± 1.85 vs. 42.13 ± 0.37 pg/mL, *p* = 0.02) and IgE (58.74 ± 0.43 vs. 45.78 ± 2.05 ng/mL, *p* = 0.003) as well as the reduction of goblet hyperplasia and subepithelial fibrosis (5 vs. 1 score, *p* = 0.001 and 5 vs. 2 score, *p* = 0.001, respectively).

**Discussion and conclusions:**

Combination of ascorbic acid with calcitriol in ineffective doses improves airway remodelling due to additive effects possibly through reduction of oxidative stress and inflammation. This study provides a scientific basis for further research and clinical applications of ascorbic acid and calcitriol and can be generalized to the broader pharmacological studies.

## Introduction

Asthma, a chronic obstructive lung disease, is characterized by the recurrent episodic symptoms including chest tightness and shortness of breath (Horak et al. 2016). This disorder affects approximately 20% of the population worldwide and exerts considerable economic burdens for either patients or healthcare systems (Ellwood et al. 2017; Nunes et al. 2017). Asthma is characterized by elevated levels of T helper (Th)-2 cytokines (e.g., interleukin (IL)-13) and immunoglobulin E (IgE) as well as inflammation (Russell and Brightling 2017). Moreover, another important characteristic of asthma is airway remodelling (i.e., goblet cell hyperplasia and subepithelial fibrosis) resulting in irreversible loss of lung function (Fahy 2015). Due to the lack of drug therapy to target airway remodelling, it remains vital to look for new therapeutic agents in asthma disease.

The exact molecular mechanisms underlying airway remodelling are poorly understood. Upon allergen exposure, Th2 cells release several cytokines such as IL-13 that finally recruit granulocytic cells resulting in inflammation (Bagnasco et al. 2016). Reactive oxygen species (ROS) produced by inflammatory cells have been reported to cause lung damage (Qu et al. 2017). Then, a repair process is initiated following injury to the airway wall. Nonetheless, dysregulation of this repair process results in airway remodelling (Humbles et al. 2004; Hirota and Martin 2013). Therefore, antioxidant agents may be effective in the prevention of asthma remodelling.

Ascorbic acid is a hydrophilic vitamin that has various beneficial effects (Chambial et al. 2013; Fukui et al. 2015). This vitamin is able to scavenge ROS and reactive nitrogen species (RNS). Thus, it prevents oxidative damage to important biological macromolecules such as DNA, lipids and proteins (Smirnoff 2018). Ascorbic acid also has anti-inflammatory activity. Moreover, some investigators have reported that this vitamin modulates immune responses (Carr and Maggini 2017). This study explores the protective effects of ascorbic acid administration on airway remodelling indices in a chronic mouse model of asthma.

Calcitriol, a lipophilic vitamin, is well-known to be important in the regulation of calcium and phosphorus homeostasis. However, the studies have indicated that this vitamin has other useful properties (Rodriguez-Lecompte et al. 2016). For example, calcitriol attenuates airway remodelling via antioxidant, anti-inflammatory and immunomodulatory effects (Penna and Adorini 2000; Kerley et al. 2015).

There are studies suggesting that a combination of antioxidants, as a result of additive or synergistic effects, may be more effective for the treatment of asthma (Tripathi et al. 2010). Thus, in this study, the protective effects of ascorbic acid in combination with calcitriol on airway remodelling were evaluated.

## Materials and methods

### Experimental design

Male wild-type BALB/c mice (6–8 weeks old) were obtained from the Department of Pharmacology, Tehran University of Medical Sciences. General protocols for animal use and experimental procedures for animal were approved by the Animal Ethics Community of Tehran University of Medical Sciences, Iran.

Before experiments, animals were acclimatized for 1 week, maintained in regular cages under the controlled environmental conditions (20 ± 2 °C and 12 h light/dark cycle) and allowed free access to standard lab chow and water. Mice were randomly divided into seven groups (*n* = 4–5) including; control group [animals were not sensitized and not challenged], asthma group [animals were sensitized and challenged by ovalbumin (OVA, grade V; Sigma, USA)], ineffective vitamin C group [asthmatic animals were orally treated with ineffective dose of ascorbic acid (Sigma, USA) (39 mg/kg in normal saline)] (Kianian et al. 2019a), ineffective vitamin D group [asthmatic animals were intraperitoneally treated with ineffective dose of calcitriol (Sigma, USA) (1.5 μg/kg in 0.9% ethanol)] (Kianian et al. 2019a), effective vitamin C group [asthmatic animals were orally treated with effective dose of ascorbic acid (130 mg/kg in normal saline)] (Kianian et al. 2019a), effective vitamin D group [asthmatic animals were intraperitoneally treated with effective dose of calcitriol (5 μg/kg in 0.9% ethanol)] (Kianian et al. 2019a), combination group [asthmatic animals were treated with the oral administration of ascorbic acid and intraperitoneal calcitriol in ineffective doses (39 mg/kg + 1.5 μg/kg)].

**Figure 1.**
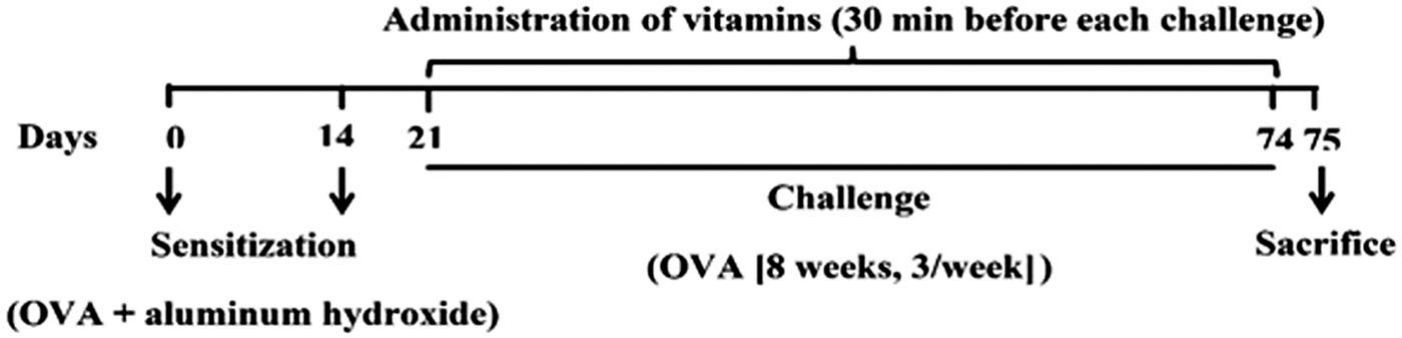
Experimental protocol of the study. OVA: ovalbumin.

For induction of a chronic model of asthma in mice, a protocol of immunization with OVA was used (Mohammadian et al. 2016, 2019; Kianmehr et al. 2016, 2017; Kianian et al. 2019a, 2019b). Briefly, except animals in the control group, others were sensitized by intraperitoneal injection of 10 µg OVA and 2 mg aluminium hydroxide (Sigma, USA) on days 0 and 14. One week later, for antigen challenge, the sensitized mice were exposed to aerosolized OVA (3% in normal saline) in a closed transparent Plexiglas chamber (dimensions 40 cm × 40 cm × 70 cm) connected to an ultrasonic nebulizer (Beurer, Germany) for 30 min once a day, three times per week for 8 consecutive weeks. In all the treatment groups, the vitamins were administrated 30 min before each challenge (Figure 1).

### Collection of bronchoalveolar lavage fluid, blood samples and lung tissues

In order to sacrifice, mice were anaesthetized by ketamine (80 mg/kg) and xylazine (8 mg/kg) intraperitoneally 24 h after the last OVA challenge. The trachea of animals was exposed and intubated with a catheter and their lungs were lavaged three times with 0.4 mL sterile normal saline. Bronchoalveolar lavage fluid (BALF) samples were transferred into microtubes and centrifuged at 1000 *g* for 5 min at 4 °C. Then the supernatants were frozen at –70 °C for further analysis. Blood was also obtained from heart, centrifuged at 3000 *g* for 10 min at 4 °C and plasma was separated to store at –70 °C for further study. The upper lobe of the right lung was resected, washed in cold saline on ice and fixed in 10% formalin for histological assessments.

### Measurements of cytokine IL-13 and IgE by enzyme-linked immunosorbent assay

The IL-13 levels in BALF supernatants and plasma IgE levels were evaluated using commercially available enzyme-linked immunosorbent assay (ELISA) kits (IBL, USA). Briefly, the ELISA plates were proportionally and step-by-step diluted. Seven standard wells were set with a volume of 225 μL each, together with blank and sample wells. Sample diluent (100 μL) and samples (50 μL) were added into the wells and gently mixed with each other (while not touching the plate walls). The plates were incubated at room temperature for 2 h, washed with buffer three times and dried on filter paper. A primary antibody working solution (100 μL) was added into each well and incubated at room temperature for 1 h. Then, the plates were washed again and 100 μL of enzyme-labelled antibody was added to each well. The plates were incubated at 37 °C for 120 min and then washed. Chromogenic substrate (100 μL) was added to each well and kept at room temperature without light for 10 min for reaction, which was terminated by adding 100 μL of stop solution into each well. Optical density (OD) was read at 450 nm by using a microplate reader (BioTek Instrument, ELX 800, Inc, USA). The OD values of the samples were plotted on semi-logarithmic paper to obtain standard curves. A zero adjustment (the OD value of the sample minus the OD value of the blank) was performed. The IL-13 and IgE concentrations were converted by the standard curve formula in accordance with the OD values of the samples.

### Lung tissue preparation for histopathology

The resected lung tissues which had been fixed in formalin were then embedded in paraffin blocks and sectioned at 4 μm thickness. Then, they were stained with periodic acid-Schiff (PAS) and Masson’s trichrome to identify goblet cell hyperplasia and subepithelial fibrosis, respectively, in five airway sections randomly for each animal. The ratio of PAS-positive cells/total cells was analyzed and their scores were calculated as follows: 0, no goblet cells; 1, <15%; 2, 15–30%; 3, 30–45%; 4, 45–60%; 5, >60% (Khakzad et al. 2012). The subepithelial fibrosis was estimated by Digimizer software. The area of collagen deposition (AC) and the perimeter of basement membrane of bronchioles (Pbm) were measured. Results were presented as the AC per Pbm (AC/Pbm μm^2^/μm). The scoring system was: 0, <5 AC/Pbm (μm^2^/μm); 1, 5–10 AC/Pbm (μm^2^/μm); 2, 10–15 AC/Pbm (μm^2^/μm); 3, 15–20 AC/Pbm (μm^2^/μm); 4, 20–25 AC/Pbm (μm^2^/μm); 5, >25 AC/Pbm (μm^2^/μm) (Cao et al. 2011). All the slides were independently scored by an expert histologist using an Olympus microscope.

### Statistical analysis

All statistical analysis was performed using SPSS software version 22.0 (SPSS Institute, Inc., USA). After evaluation of data normalization, a one-way analysis of variance (ANOVA) followed by Tukey’s post hoc was used for quantitative comparisons among groups and data were presented as the mean ± standard error of the mean (SEM). Kruskal–Wallis analysis of variance was used to analyze histology scores and data were presented as median values. *p* values <0.05 were regarded statistically significant.

## Results

### Effect of ascorbic acid and calcitriol and their combination on IL-13 levels in BALF supernatants

**Figure 2.**
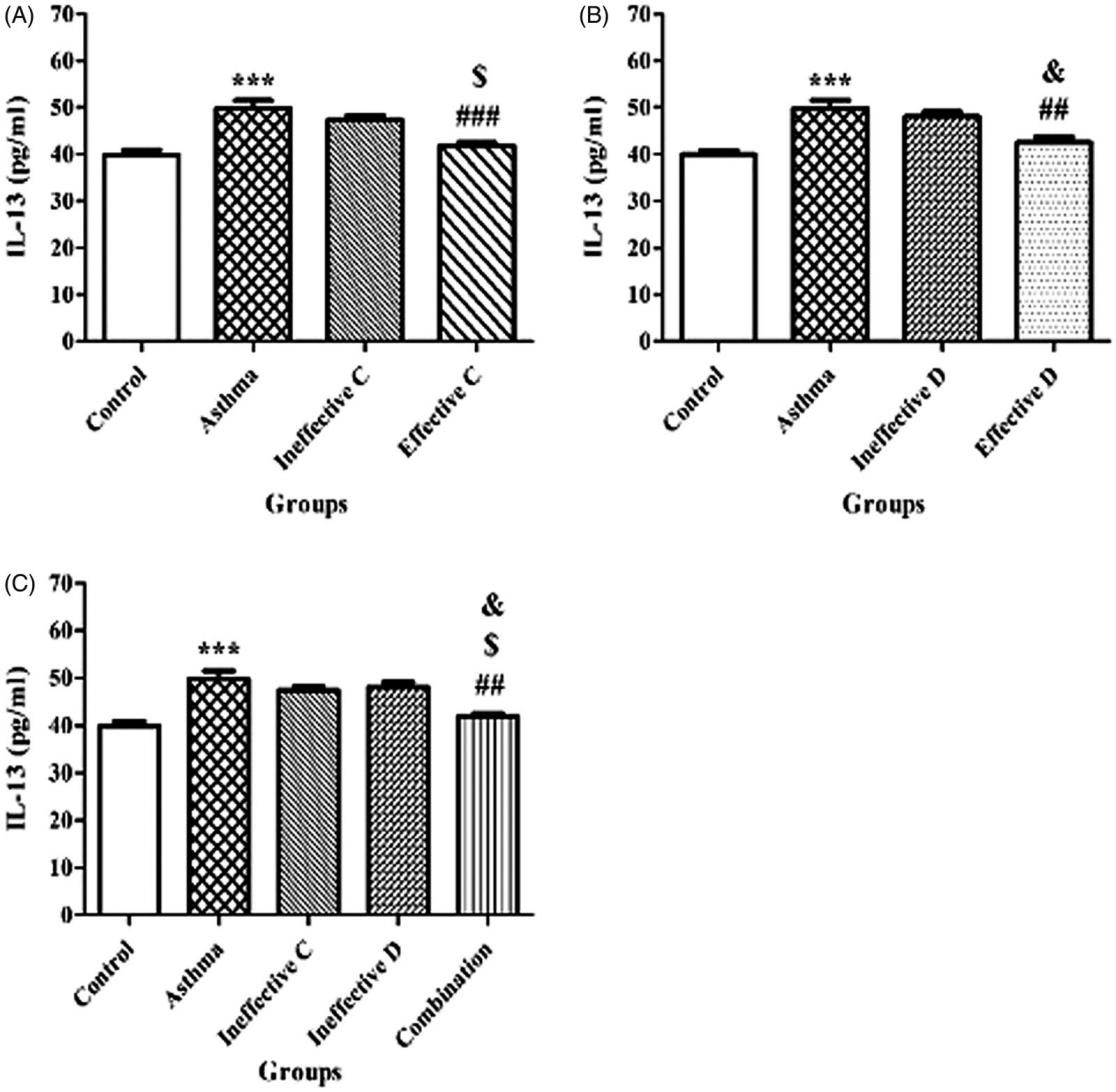
Changes of interleukin (IL)-13 levels in bronchoalveolar lavage fluid supernatants with ineffective and effective doses of ascorbic acid (A), with ineffective and effective doses of calcitriol (B) and with combination of ineffective doses of ascorbic acid and calcitriol (C). Data are expressed as mean ± SEM. ***p < 0.001 versus the control group. ^##^*p* < 0.01 versus the asthma group. ^###^*p* < 0.001 versus the asthma group. ^$^*p* < 0.05 versus the ineffective C group. ^&^*p* < 0.05 versus the ineffective D group. Ineffective C: ascorbic acid with ineffective dose; Ineffective D: calcitriol with ineffective dose; Effective C: ascorbic acid with effective dose; Effective D: calcitriol with effective dose.

There was a significant increase in IL-13 levels in the asthma group compared to the control group (50.5 ± 1.85 vs. 40.13 ± 0.31 pg/mL, *p* = 0.000) (Figure 2). Administration of ascorbic acid and calcitriol in effective doses significantly decreased IL-13 levels in comparison with the asthma group (42.73 ± 0.27 vs. 50.5 ± 1.85 pg/mL, *p* = 0.009 and 43.24 ± 0.37 vs. 50.5 ± 1.85 pg/mL, *p* = 0.04, respectively) (Figure 2). There were no significant differences in IL-13 levels in the groups of ascorbic acid and calcitriol in ineffective doses compared to the asthma group (47.93 ± 0.29 vs. 50.5 ± 1.85 pg/mL and 48.14 ± 0.47 vs. 50.5 ± 1.85 pg/mL, respectively) (Figure 2). However, combined administration of ascorbic acid and calcitriol in ineffective doses significantly decreased IL-13 levels in comparison with the asthma group (42.13 ± 0.37 vs. 50.5 ± 1.85 pg/mL, *p* = 0.02) (Figure 2).

### Effect of ascorbic acid and calcitriol and their combination on plasma IgE levels

**Figure 3.**
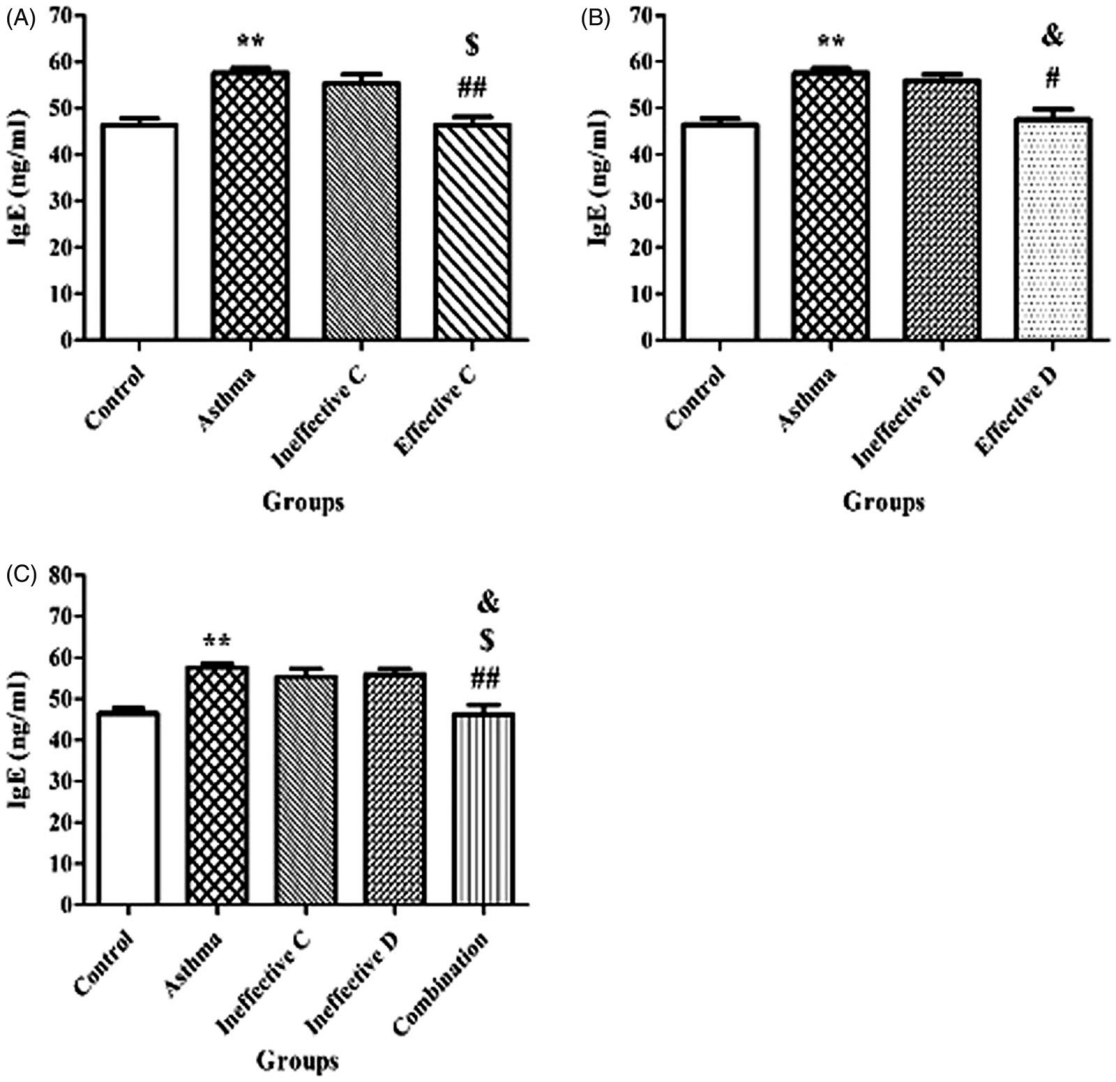
Changes in plasma immunoglobulin (Ig) E levels with ineffective and effective doses of ascorbic acid (A), with ineffective and effective doses of calcitriol (B) and with combination of ineffective doses of ascorbic acid and calcitriol (C). Data are expressed as mean ± SEM. **p < 0.01 versus the control group. ^#^*p* < 0.05 versus the asthma group. ^##^*p* < 0.01 versus the asthma group. ^$^*p* < 0.05 versus the ineffective C group. ^&^*p* < 0.05 versus the ineffective D group. Ineffective C: ascorbic acid with ineffective dose; Ineffective D: calcitriol with ineffective dose; Effective C: ascorbic acid with effective dose; Effective D: calcitriol with effective dose.

There was a significant increase in IgE levels in the asthma group compared to the control group (58.74 ± 0.43 vs. 46.28 ± 0.85 ng/mL, *p* = 0.004) (Figure 3). Administration of ascorbic acid and calcitriol in effective doses significantly decreased IgE levels in comparison with the asthma group (46.17 ± 1.55 vs. 58.74 ± 0.43 ng/mL, *p* = 0.002 and 46.77 ± 1.62 vs. 58.74 ± 0.43 ng/mL, *p* = 0.01, respectively) (Figure 3). There were no significant differences in IgE levels in the groups of ascorbic acid and calcitriol in ineffective doses compared to the asthma group (56.64 ± 1.82 vs. 58.74 ± 0.43 ng/mL and 57.13 ± 1.29 vs. 58.74 ± 0.43 ng/mL, respectively) (Figure 3). However, combined administration of ascorbic acid and calcitriol in ineffective doses decreased IgE levels in comparison with the asthma group significantly (45.78 ± 2.05 vs. 58.74 ± 0.43 ng/mL, *p* = 0.003) (Figure 3).

### Effect of ascorbic acid and calcitriol and their combination on goblet cell hyperplasia

**Figure 4.**
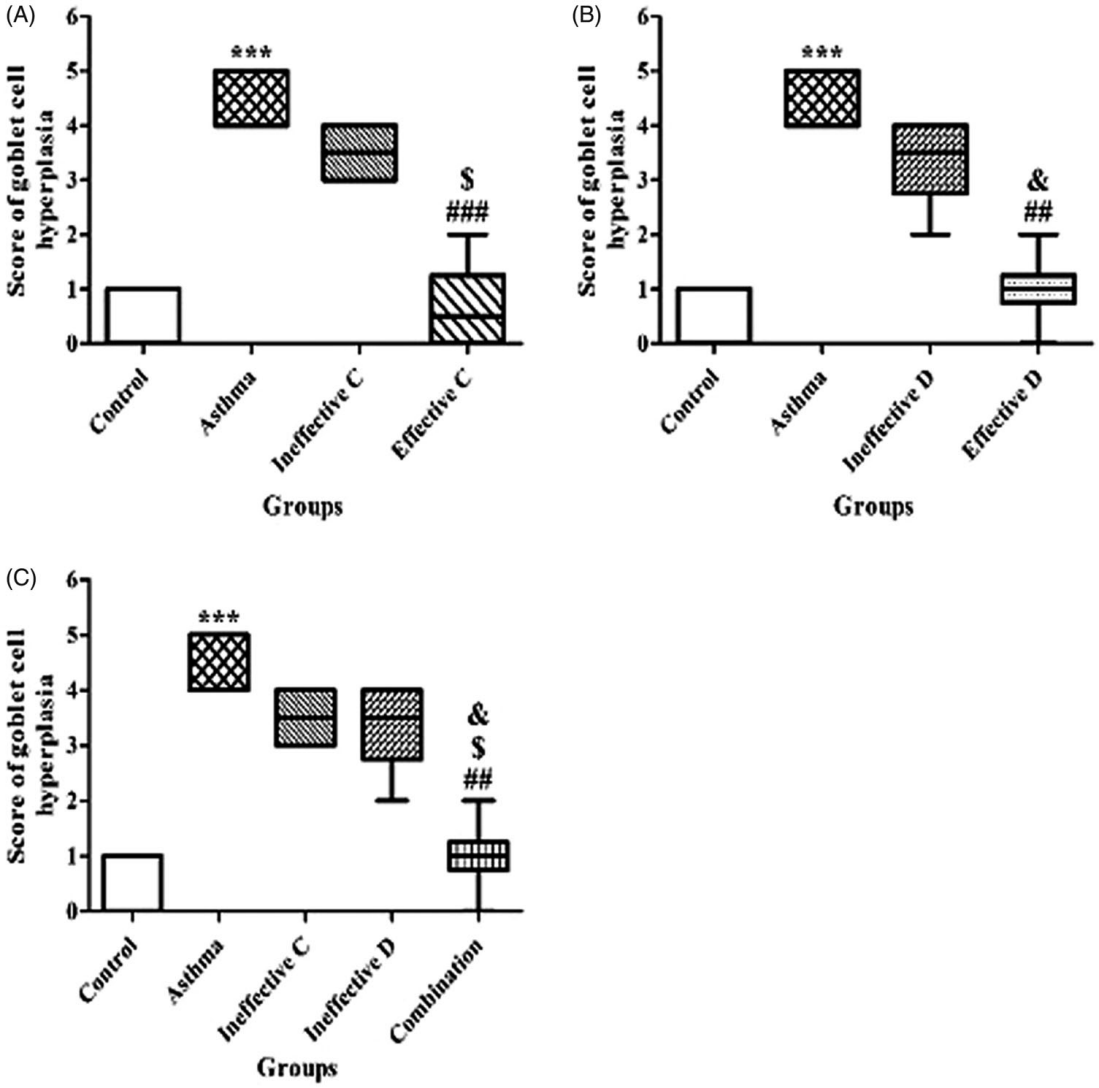
The goblet cell hyperplasia score with ineffective and effective doses of ascorbic acid (A), with ineffective and effective doses of calcitriol (B) and with combination of ineffective doses of ascorbic acid and calcitriol (C). Data are expressed as median values. ***p < 0.001 versus the control group. ^##^*p* < 0.01 versus the asthma group. ^###^*p* < 0.001 versus the asthma group. ^$^*p* < 0.05 versus the ineffective C group. ^&^*P* < 0.05 versus the ineffective D group. Ineffective C: ascorbic acid with ineffective dose; Ineffective D: calcitriol with ineffective dose; Effective C: ascorbic acid with effective dose; Effective D: calcitriol with effective dose.

**Figure 5.**
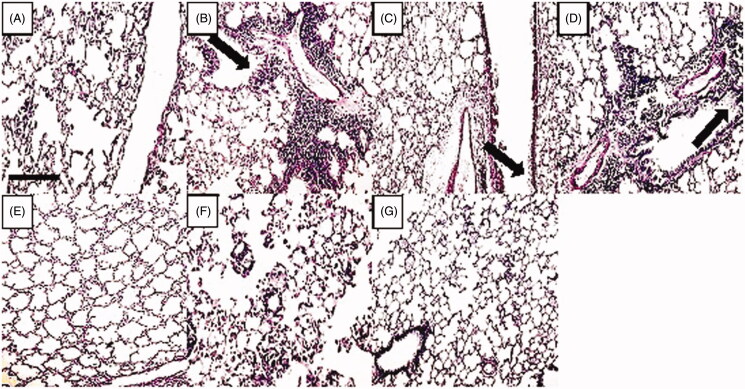
Changes in lung histology in different groups with periodic acid-Schiff (PAS) staining (light microscopy, magnification 20×). (A): control, (B): asthma, (C): ineffective C, (D): ineffective D, (E): effective C, (F): effective D and (G): combination groups. In the rats of the control group (A), there was no goblet cell hyperplasia. In the asthma group (B), there was a significant increase in goblet cell hyperplasia. Administration of ascorbic acid and calcitriol in ineffective doses did not improve goblet cell hyperplasia (C and D, respectively). However, administration of ascorbic acid and calcitriol in effective doses, as well as combination of both vitamins in ineffective doses, decreased goblet cell hyperplasia. Arrows show goblet cell hyperplasia. Bar: 100 μm.

There was a significant increase in the score of PAS-positive goblet cells in the asthma group compared to the control group (5 vs. 1 score, *p* = 0.000) (Figure 4). Administration of ascorbic acid and calcitriol in effective doses significantly decreased the score of PAS-positive goblet cells in comparison with the asthma group (1 vs. 5 score, *p* = 0.000 and 1 vs. 5 score, *p* = 0.001, respectively) (Figure 4). There were no significant differences in the score of PAS-positive goblet cells in the groups of ascorbic acid and calcitriol in ineffective doses compared to the asthma group (4 vs. 5 score and 4 vs. 5 score, respectively) (Figure 4). However, combined administration of ascorbic acid and calcitriol in ineffective doses decreased the score of PAS-positive goblet cells in comparison with the asthma group significantly (1 vs. 5 score, *p* = 0.001) (Figure 4). Lung histopathology photographs in different groups were also provided (Figure 5).

### Effect of ascorbic acid and calcitriol and their combination on subepithelial fibrosis

**Figure 6.**
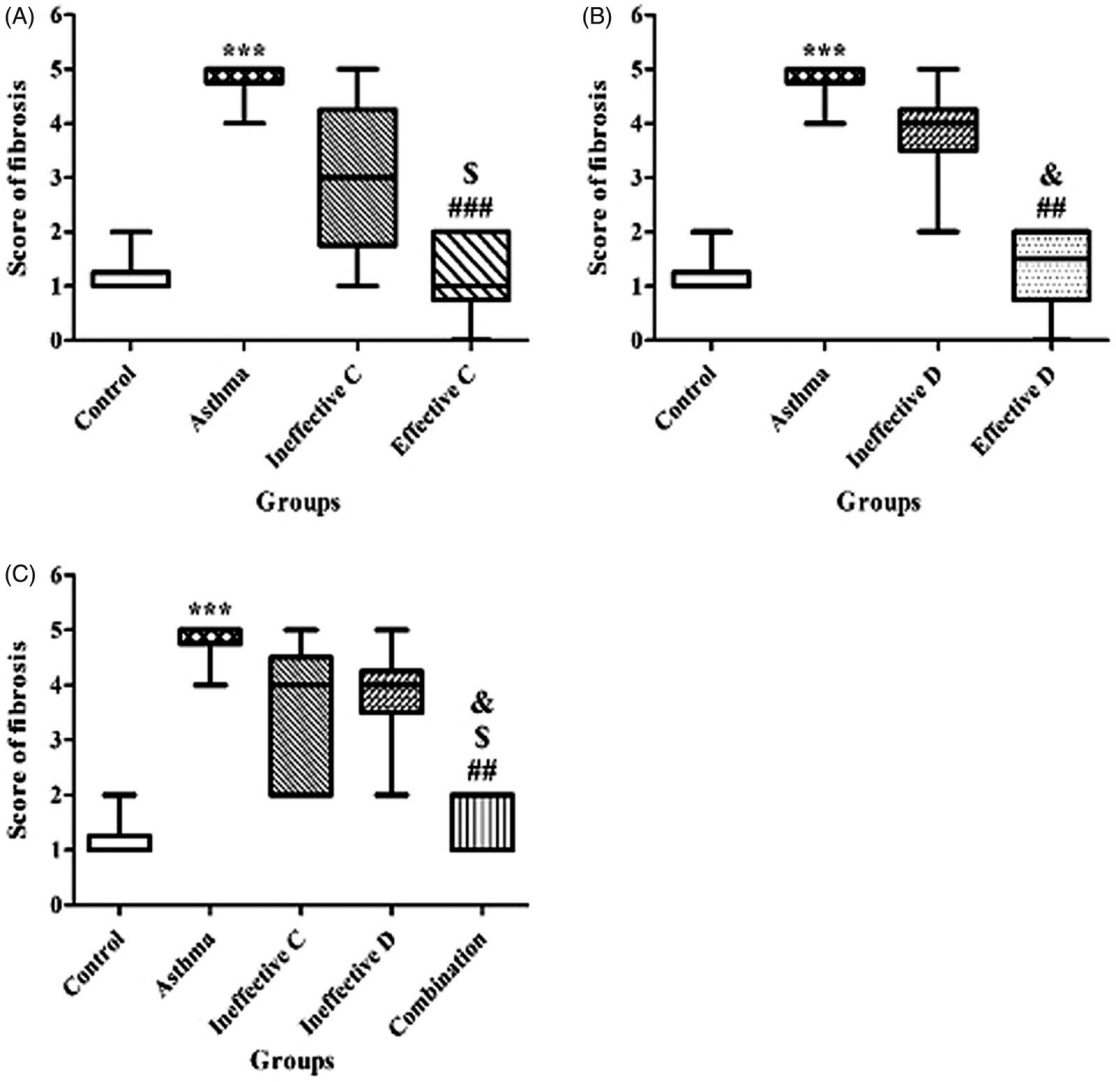
The subepithelial fibrosis score with ineffective and effective doses of ascorbic acid (A), with ineffective and effective doses of calcitriol (B) and with combination of ineffective doses of ascorbic acid and calcitriol (C). Data are expressed as median values. ***p < 0.001 versus the control group. ^##^*p* < 0.01 versus the asthma group. ^###^*p* < 0.001 versus the asthma group. ^$^*p* < 0.05 versus the ineffective C group. ^&^*p* < 0.05 versus the ineffective D group. Ineffective C: ascorbic acid with ineffective dose; Ineffective D: calcitriol with ineffective dose; Effective C: ascorbic acid with effective dose; Effective D: calcitriol with effective dose.

**Figure 7.**
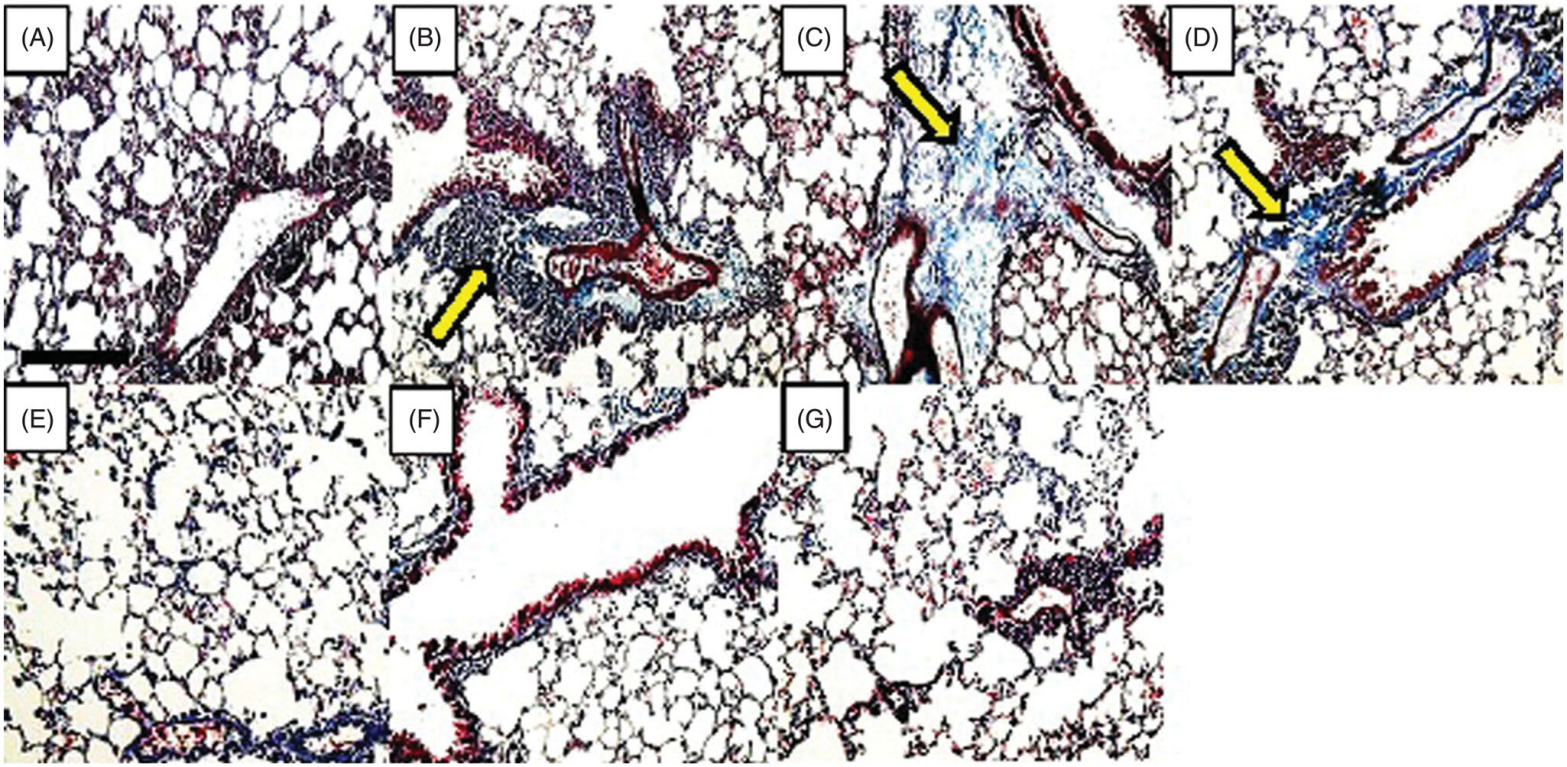
Changes in lung histology in different groups with Masson’s trichrome staining (light microscopy, magnification 20×). (A): control, (B): asthma, (C): ineffective C, (D): ineffective D, (E): effective C, (F): effective D and (G): combination groups. In the rats of the control group (A), there was no collagen deposition. In the asthma group (B), there was a significant increase in collagen deposition. Administration of ascorbic acid and calcitriol in ineffective doses did not improve collagen deposition (C and D, respectively). However, administration of ascorbic acid and calcitriol in effective doses, as well as combination of both vitamins in ineffective doses, decreased collagen deposition. Arrows show collagen deposition. Bar: 100 μm.

There was a significant increase in the score of subepithelial fibrosis in the asthma group compared to the control group (1 vs. 5 score, *p* = 0.000) (Figure 6). Administration of ascorbic acid and calcitriol in effective doses significantly decreased the score of subepithelial fibrosis in comparison with the asthma group (2 vs. 5 score, *p* = 0.000 and 2 vs. 5 score, *p* = 0.001, respectively) (Figure 6). There were no significant differences in the score of subepithelial fibrosis in the groups of ascorbic acid and calcitriol in ineffective doses compared to the asthma group (4 vs. 5 score and 4 vs. 5 score, respectively) (Figure 6). However, combined administration of ascorbic acid and calcitriol in ineffective doses decreased the score of subepithelial fibrosis in comparison with the asthma group significantly (2 vs. 5 score, *p* = 0.001) (Figure 6). Lung histopathology photographs in different groups were also provided (Figure 7).

## Discussion

In the experimental studies, one of the most extensively used models for evaluation of the asthma pathogenesis is the murine OVA sensitization and challenge model (Janssen-Heininger et al. 2009).

Airway remodelling is an important characteristic of asthma that its exact molecular mechanisms are not fully elucidated (Kwak et al. 2015). Nevertheless, several causal factors have shown to contribute to the development of airway remodelling. Upon allergen exposure, innate immune cells (e.g., dendritic cells) process it and inform naive Th cells about invading pathogens contributing to the initial commitment of naive Th cells into Th2 subsets (Hirose et al. 2017). Th2 cells release various cytokines including IL-13 which induce IgE production from B cells (Bagnasco et al. 2016). Then, IgE binds to its receptors on mast cells and basophils and recruits granulocytic cells leading to inflammation (Froidure et al. 2016). These granulocytic cells, particularly eosinophils, are the major source of ROS as the main cause of oxidative damage to the biological molecules such as lipids resulting in tissue injury (Qu et al. 2017). Then, repair process is initiated in response to injury to the airway wall. However, dysregulation of this repair process leads to airway remodelling (Humbles et al. 2004; Hirota and Martin 2013).

An immunoregulatory cytokine promotes inflammatory processes and induces structural changes to the airways is IL-13 (Fehrenbach et al. 2017). The present study demonstrated that IL-13 levels in BALF supernatants were significantly higher in the asthma group compared to the control group. This observation is in accordance with the studies of Mohammadian et al. (2019) that reported an increase in IL-13 levels with asthma induction.

IgE is another important cytokine that plays a key role in the propagation of airway inflammation and an increased IgE production is the strongest predisposing factor for the development of asthma (Skiepko et al. 2009). In this study, OVA-induced asthma caused a significant increase of plasma IgE levels. This finding is in good agreement with Liu et al. (2009) study that found a considerable increase in serum levels of total IgE in OVA-challenged mice.

Goblet cell hyperplasia and subepithelial fibrosis are two characteristics of airway remodelling (Samitas et al. 2018). In this line, we also observed that in the asthma group, there were goblet cell hyperplasia and subepithelial fibrosis compared to the control group.

**Figure 8.**
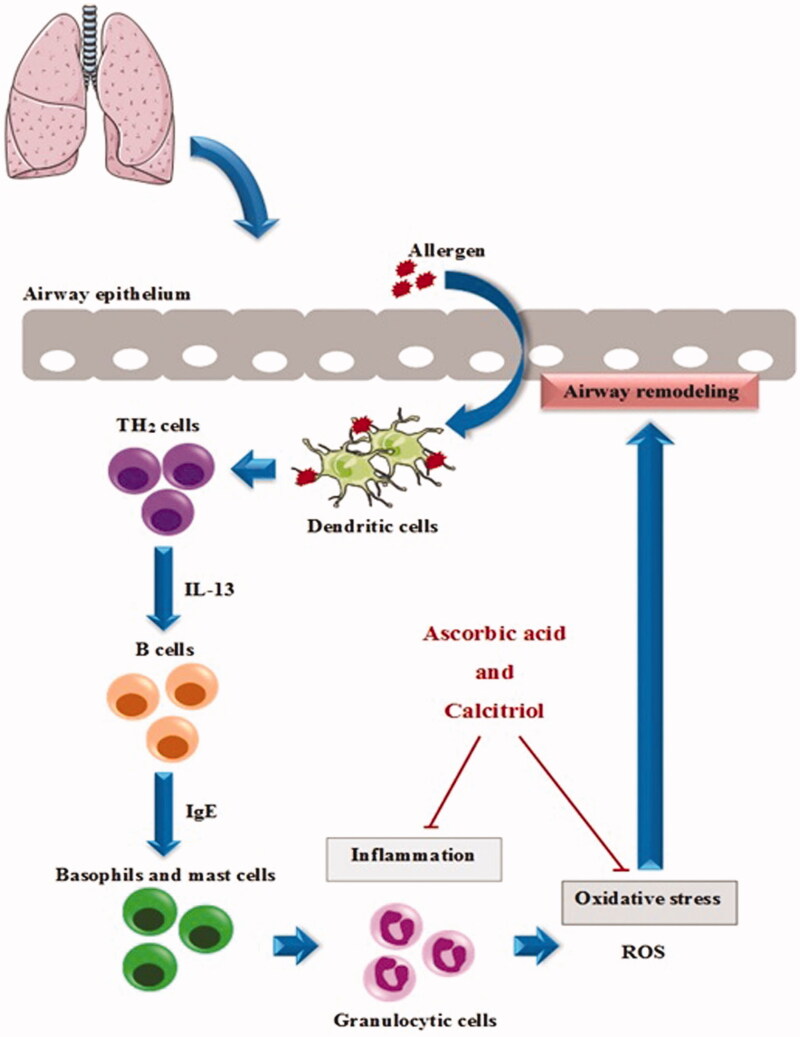
Schematic illustration of the protective effects of ascorbic acid and calcitriol and their combination in attenuating airway remodelling. Ascorbic acid and calcitriol independently are capable of decreasing inflammation and oxidative stress in asthma disease resulting in the reduction of airway remodelling. Combined administration of ascorbic acid and calcitriol in ineffective doses also mitigates airway remodelling due to additive effects probably through reduction of oxidative stress and inflammation.

Ascorbic acid is a water-soluble vitamin that contributes to antioxidant activity by scavenging ROS and reactive nitrogen species resulting in the prevention of oxidative damage to important biological macromolecules such as DNA, lipids and proteins (Smirnoff 2018; Kianian et al. 2019a). In addition to antioxidative effects, ascorbic acid is also known to modulate immune responses (Carr and Maggini 2017). However, two independent studies have reported some side effects associated with ascorbic acid in other organs including nausea, diarrhea and nephropathy (Sestili 1983; Lin et al. 2019). According to beneficial properties of ascorbic acid, it might be a good candidate to prevent airway remodelling during asthma. In this study, administration of ascorbic acid in effective dose significantly decreased levels of IL-13 and IgE compared to the asthma group. Similar to our results, a couple of studies have shown that administration of ascorbic acid modulates T cell proliferation and cytokine secretion and reduces IgE (Noh et al. 2005; Maeng et al. 2009). Moreover, in the current study, ascorbic acid administration in effective dose was able to decrease goblet cell hyperplasia and subepithelial fibrosis. The protective effects of ascorbic acid have been summarized in Figure 8.

Calcitriol is a fat-soluble vitamin known to be involved in mineral and skeletal homeostasis (Veldurthy et al. 2016). However, it has been suggested that this vitamin may be a potent antioxidant (Mokhtari et al. 2017; Kianian et al. 2019a). In addition, calcitriol exerts other varieties of biological effects in the immune system such as inhibiting T cell proliferation and controlling the expression of cytokines (Mora et al. 2008). Nevertheless, calcitriol administration may cause side effects in other organs such as nausea, vomiting, hypercalciuria, nephrocalcinosis and secondary hyperparathyroidism (Makitie et al. 2003). Considering the valuable effects of calcitriol, it seems reasonable to study the effects of calcitriol on prevention of the airway remodelling in asthma. In this study, calcitriol administration in effective dose significantly attenuated increases in levels of IL-13 and IgE compared to the asthma group. Similarly, the study by Zhong et al. (2013) showed that calcitriol decreased IL-13 levels in the culture supernatant compared with lipopolysaccharide-stimulated alone group. In a study by Hartmann et al. (2011), this vitamin reduced the production of IgE from peripheral human B cells. In the present study, calcitriol administration in effective dose reduced goblet cell hyperplasia and subepithelial fibrosis. Similarly, Lai et al. (2013) reported that calcitriol could protect OVA sensitized mice from airway remodelling. The protective effects of ascorbic acid have been summarized in Figure 8.

There are various studies suggesting that a combination of antioxidants may be more effective than the individuals, owing to additive or synergistic effects (Nounou et al. 2010). In this line, our study found that administration of ascorbic acid in combination with calcitriol in ineffective doses was also capable to decrease the levels of IL-13 and IgE as well as goblet cell hyperplasia and subepithelial fibrosis. The protective effects of ascorbic acid have been summarized in Figure 8.

## Conclusions

The present study demonstrates that ascorbic acid mitigates airway remodelling. In addition, although ascorbic acid and calcitriol in ineffective doses individually do not exert any protective effects, combination of both vitamins causes attenuation of airway remodelling due to additive effects possibly through reduction of oxidative stress and inflammation.
